# Grounding Soil Carbon Sequestration Claims: The Role of Long‐Term EC Flux Data

**DOI:** 10.1111/gcb.70439

**Published:** 2025-08-18

**Authors:** Elaine Mitchell, David Rowlings, Jonathan Sanderman

**Affiliations:** ^1^ Queensland University of Technology Brisbane Queensland Australia; ^2^ Woodwell Climate Research Centre Falmouth Massachusetts USA

**Keywords:** cropping, EC flux, long‐term SOC monitoring, net C sink or source, net ecosystem carbon balance, soil organic carbon

Soil is the largest terrestrial reservoir of organic carbon and plays a critical role in regulating the global C cycle. Increasing soil organic carbon (SOC) sequestration not only supports productivity and resilience but also draws down atmospheric CO_2_, offering a potential natural climate solution. As such, SOC management features prominently in national targets, corporate sustainability commitments, and carbon accounting protocols. These efforts typically rest on three widely accepted assumptions articulated by Matson et al. (1997) that: (a) the conversion of native vegetation to cropland results in substantial SOC depletion; (b) SOC stocks stabilize under conventional management at a new, lower equilibrium; and (c) improved practices, such as reduced or no‐tillage, can incrementally restore SOC stocks, repaying the C debt incurred through land‐use change.

While assumption (a) is well supported across diverse agricultural systems and climates, Moore et al. (2025) challenge the validity of assumptions (b) and (c) using over a decade of high‐resolution eddy covariance (EC) flux measurements across multiple cropping systems in the U.S. Midwest. Their analysis of net ecosystem carbon balance (NECB) reveals that conventionally tilled annual cropping systems continue to act as net C sources, no‐till systems are approximately C neutral, and only perennial‐based systems demonstrate consistent C storage.

The significance of this study lies not only in its findings but in its methodology. Most research on SOC change in agricultural systems relies on plot‐scale measurements with limited spatial and temporal replication, often based on comparisons between baseline and re‐sampled soil cores. Apparent SOC increases under no‐till, for example, may reflect redistribution within the profile rather than net gains (e.g., Cai et al. 2022). Additionally, many studies conflate SOC concentration with SOC stock, with the latter requiring accurate bulk density measurements. This is especially critical in no‐till systems, where bulk density can change over time and influence stock estimates (Powlson et al. 2014).

To address these limitations, ecosystem‐scale C flux measurements provide a valuable complementary methodology. Moore et al. (2025) employ long‐term, decadal‐scale EC flux monitoring—widely considered the gold standard for quantifying whole‐ecosystem C exchange (Baldocchi 2020)—to produce continuous, standardized, high‐resolution data on NECB at the field scale. Unlike pool‐based accounting, which infers change from snapshots of large and spatially variable SOC stocks, EC flux captures dynamic C inputs and outputs over time, revealing whether a system is acting as a net C source or sink.

Drawing on 7–17 years of EC data, the authors quantify net ecosystem exchange (NEE), net ecosystem productivity (NEP), and, crucially, net ecosystem C balance (NECB), which accounts for C removed during harvest. The study spans multiple cropping systems, including conventionally tilled maize‐soybean rotations, no‐till systems, and perennial systems such as *Miscanthus × giganteus*, switchgrass ( *Panicum virgatum* ), and restored tallgrass prairie.

The authors find no evidence that soils in long‐established annual cropping systems have reached steady‐state. Despite over a century of continuous cropping, conventionally tilled maize–soybean fields remain persistent net sources of C—challenging assumption (b). Similarly, while no‐till slows C loss relative to conventional tillage, it does not accumulate SOC—challenging assumption (c). This suggests that while no‐till provides agronomic benefits such as erosion control and yield stability, it may not reliably remove CO_2_ from the atmosphere.

In contrast, perennial systems such as Miscanthus, switchgrass, and restored tallgrass prairie acted as consistent C sinks, exhibiting negative NECB values. Notably, these systems began accumulating C within the first year of establishment, underscoring their relatively rapid and sustained sequestration potential. Highly productive perennial grass monocultures (particularly Miscanthus and switchgrass) are especially promising due to their high allocation to belowground biomass, which enhances SOC storage and improves drought resilience (Blakely et al. 2025).

The findings of Moore et al. (2025) highlight a critical distinction in nature‐based agricultural climate solutions: the difference between emission reduction practices that slow SOC losses relative to a business‐as‐usual baseline and C removal practices that result in net atmospheric CO_2_ sequestration. In the case of interventions like no‐till (where SOC may stabilise but not necessarily increase) the study emphasizes the need for dynamic baselines and well‐defined counterfactuals in carbon accounting frameworks. To illustrate, the Australian Carbon Credit Unit (ACCU) scheme (Soil Carbon Method 2021) currently applies a static baseline that assumes SOC would remain stable in the absence of intervention. However, if the actual business‐as‐usual scenario involves ongoing SOC decline, this static approach may underestimate the full mitigation benefit of improved management.

The finding that no‐till may not actively draw down atmospheric CO_2_ should not deter its continued adoption. In contemporary soil–climate discussions, C sequestration is often treated as the primary objective while other benefits such as improved water retention, reduced erosion, and enhanced food security are considered secondary. This narrow framing is potentially misguided. As Amundson and Biardeau (2018) argue, SOC management should be embedded within broader strategies aimed at strengthening agricultural resilience and sustainability in the face of a changing climate.

While Moore et al. (2025) acknowledge that their results are derived from one region, they suggest that the observed trends are likely broadly applicable to other cropping systems globally. We agree that the study provides robust evidence on the *direction* of C change under different management regimes. However, caution is warranted when extrapolating the *magnitude* of these findings, particularly given the presence of artificial tile drainage at the study site. Tile drainage lowers the water table, improving soil aeration and accelerating microbial decomposition of SOC. It is therefore unsurprising that relatively high C losses (−2.6 t C ha^−1^ year^−1^) were observed under a low‐input, conventionally tilled system with a moderately high initial SOC level (1.5%–2%). The influence of artificial drainage on SOC stocks has been documented by Jacinthe et al. (2001) who found that undrained cropping systems contained 15% more SOC than comparable tile‐drained systems.

In extrapolating findings, we must also consider fluxes that are not captured by the EC flux approach. Based on a preliminary literature review, we provide indicative estimates of losses that may be missing from NECB calculations. First, EC does not detect lateral C flows, such as organic C in subsurface drainage, which may be enhanced by tile drainage infrastructure. That said, the magnitude of this loss pathway appears small relative to the reported NECB. For example, Royer and David (2005) measured dissolved organic C losses via tile drains in the U.S. Midwest at just 0.02 t C ha^−1^ year^−1^. Second, NECB does not account for non‐CO_2_ greenhouse gas emissions, such as nitrous oxide (N_2_O), which could partially offset gains in SOC. McGowan et al. (2019), for instance, reported average N_2_O emissions equivalent to 0.32 t C ha^−1^ year^−1^ in miscanthus and switchgrass systems. Third, C losses from insect and pest herbivory are also excluded from NECB calculations. In U.S. corn systems, estimated yield losses to invertebrate pests (insects, mites, slugs) are around 7% (Musser et al. 2023), which equates to a C loss of approximately 0.35 t C ha^−1^ year^−1^, assuming an average yield of 11 t ha^−1^. This rate is likely to be much lower in perennial systems such as miscanthus and switchgrass, which appear to experience minimal insect herbivory (Nabity et al. 2011). Together, these unaccounted losses could amount to ~0.7 t C ha^−1^ year^−1^. While this estimate is coarse, it underscores the importance of interpreting NECB values in the context of fluxes that lie outside of the EC flux measurement envelope.

Given that no single method can fully capture both the direction and magnitude of C change in agricultural systems, there is growing consensus on the need to generate coordinated, long‐term monitoring networks. These networks should integrate complementary approaches, including EC flux towers, non‐CO_2_ gas measurements, soil and biomass inventories, high‐resolution satellite data, and near‐surface sensing technologies (e.g., Paustian et al. 2019).

Despite the growing consensus on the value of integrated monitoring, flux tower coverage in managed systems remains limited due to cost. However, advances in low‐cost sensor technologies may reduce barriers and expand deployment. Strategically designed networks—spanning key agro‐ecological zones and incorporating paired EC flux towers (i.e., conventional versus improved management)—are essential for enabling robust causal inference at commercial scales. Such a design strengthens the ability to attribute changes in C dynamics to specific management interventions and generates high‐quality datasets needed to validate and refine process‐based models, and contribute to robust C monitoring, reporting, and verification (MRV) frameworks. In Australia, this rationale underpins the development of the Australian Long‐Term Agroecosystem Research (ALTAR) network, which has deployed paired flux towers across representative bioregions.

If agriculture is to make a credible contribution to climate change mitigation, expectations must be grounded in robust, long‐term empirical evidence—such as the data presented by Moore et al. (2025). Their findings underscore the importance of using real‐world, decadal‐scale observations to inform C accounting frameworks and investment decisions. Such studies provide essential ‘guard rails’ –helping to align policy, practice, and finance with what is scientifically and operationally achievable. In doing so, they ensure that SOC initiatives are not only ambitious but also credible, durable, and grounded in ecological reality.

## Conflicts of Interest

The authors declare no conflicts of interest.

## Linked Articles

This article is a Invited Commentary on Moore et al., https://doi.org/10.1111/gcb.70291.

## Data Availability

Data sharing not applicable to this article as no datasets were generated or analysed for the current article.
